# Mapping the Binding Interface between an HIV-1 Inhibiting Intrabody and the Viral Protein Rev

**DOI:** 10.1371/journal.pone.0060259

**Published:** 2013-04-02

**Authors:** Thomas Vercruysse, Eline Boons, Tom Venken, Els Vanstreels, Arnout Voet, Jan Steyaert, Marc De Maeyer, Dirk Daelemans

**Affiliations:** 1 Rega Institute for Medical Research, KU Leuven, Leuven, Belgium; 2 Division of Biochemistry, Molecular and Structural Biology, Department of Chemistry, KU Leuven, Leuven, Belgium; 3 Structural Biology Brussel Laboratory, Department of Molecular Interactions, Vrije Universiteit Brussel, Brussels, Belgium; George Mason University, United States of America

## Abstract

HIV-1 Rev is the key protein in the nucleocytoplasmic export and expression of the late viral mRNAs. An important aspect for its function is its ability to multimerize on these mRNAs. We have recently identified a llama single-domain antibody (Nb_190_) as the first inhibitor targeting the Rev multimerization function in cells. This nanobody is a potent intracellular antibody that efficiently inhibits HIV-1 viral production. In order to gain insight into the Nb_190_-Rev interaction interface, we performed mutational and docking studies to map the interface between the nanobody paratope and the Rev epitope. Alanine mutants of the hyper-variable domains of Nb_190_ and the Rev multimerization domains were evaluated in different assays measuring Nb_190_-Rev interaction or viral production. Seven residues within Nb_190_ and five Rev residues are demonstrated to be crucial for epitope recognition. These experimental data were used to perform docking experiments and map the Nb_190_-Rev structural interface. This Nb_190_-Rev interaction model can guide further studies of the Nb_190_ effect on HIV-1 Rev function and could serve as starting point for the rational development of smaller entities binding to the Nb_190_ epitope, aimed at interfering with protein-protein interactions of the Rev N-terminal domain.

## Introduction

Nuclear export of viral mRNAs is a crucial step in the HIV-1 replication cycle [1]. Fully spliced mRNA expressing the ‘early genes’ is exported through the cellular host mechanism. In contrast, for the transport of unspliced and incompletely spliced ‘late’ mRNA species that encode structural viral proteins and serve as viral RNA genome, HIV-1 uses a complex mechanism. These late viral RNA species all contain a secondary structured RNA element (Rev responsive element or RRE) on which a multimeric Rev export complex is formed [2], [3] that employs the CRM1-mediated cellular pathway for nuclear export [4]–[6].

**Figure 1:**
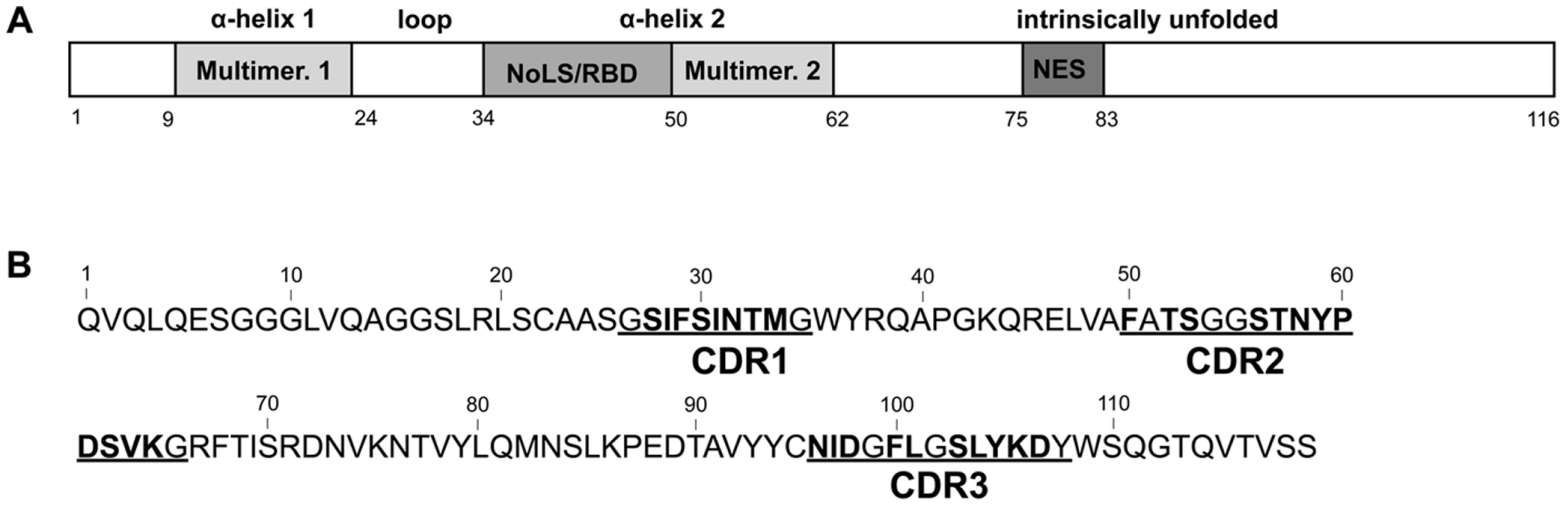
Rev and Nb_190_ protein organization and mutation scheme. (**A**) Schematic representation of the HIV-1 Rev functional domain organization and secondary structures. The N-terminal domain of Rev forms a helix-loop-helix while de C-terminal domain is intrinsically unfolded. Three functional domains are shown: the Nuclear Export Signal (NES), the Nucleolar Localization Signal (NoLS) that also serves as RNA Binding Domain (RBD) and the first and second multimerization domain (Multimer. 1 and Multimer. 2). (**B**) Mutation scheme of the Nb_190_ alanine scan. The three hyper-variable CDR regions are underlined as defined by the IMGT [50]. Residues that were mutated to alanine are shown in bold.

The HIV-1 Rev protein consists of 116 amino acids (Fig. 1*A*). The N-terminal helix-turn-helix loop [7] contains a basic arginine-rich stretch that interacts with the RRE and also serves as a nucleolar localization signal (NoLS) [2]. This NoLS is flanked by two hydrophobic regions responsible for Rev multimerization [8]. In the C-terminal part of the protein a leucine-rich nuclear export signal is located that interacts with CRM1 [4]–[6], [9], [10]. Although under steady state conditions Rev localizes mainly to the nucleoli, it shuttles continuously between the cytoplasm and the nucleus [11], [12].

There have been several attempts to inhibit the crucial function of Rev, mostly targeting the Rev-RRE and Rev-CRM1 interactions; reviewed in [13]. A third aspect of the Rev function is its requirement for multimerization. Multimerization of Rev has been demonstrated both *in vitro* and in cell culture [14], [15]. Initial binding to the high-affinity Rev binding site of the RRE (stem-loop IIB) is followed by multimerization of Rev along the RRE template *via* a combination of cooperative hydrophobic protein-protein interactions and electrostatic protein-RNA interactions leading to further coating of stem IIA and stem I of the RRE [3], [16], [17]. The two α-helical multimerization regions of Rev combine into a dimerization (tail) and a multimerization (head) surface allowing the formation of Rev multimers through tail-tail and head-head interactions [15], [18], [19]. Due to aggregation properties of Rev at high concentrations in solution, the structure determination of Rev has been hampered for a long time. Recently, this Rev structure has been elucidated using a multimerization deficient mutant [18] and a monoclonal Fab fragment inhibiting the Rev multimerization [19]. However, monoclonal Fab fragments are not easily amenable for intracellular expression and have therefore limited applications for inhibiting the Rev multimerization inside living cells. We have recently discovered a single-domain nanobody (Nb_190_) as the first entity that interferes with Rev multimerization and potently inhibits HIV-1 production inside cells [20]. Nanobodies are derived from heavy-chain antibodies of *Camelidae*. They are small, highly soluble, single gene entities fully capable of recognizing specific epitopes [21], [22]. Nb_190_ binds to the head multimerization surface of Rev thereby hindering its multimerization both *in vitro* and in cell culture. Interestingly, this antibody is fully functional inside a cellular environment and is able to interact with Rev inside an infected cell causing a strong reduction in HIV-1 production. These observations raise the question to what extent targeting the Rev multimerization surfaces could contribute to improved antiviral therapy. Therefore we aimed at identifying the individual paratope and epitope residues crucial for Rev recognition by Nb_190_. Based on these mutational data, we performed docking studies to create a detailed view on the Nb_190_-Rev protein-protein interaction interface.

## Materials and Methods

### Cell Culture, Transfections and Plasmids

Prokaryotic and eukaryotic expression vectors were constructed using standard molecular cloning techniques. pRev-AcGFP expresses the Rev protein fused to the monomeric Aequorea coerulescens green fluorescent protein (AcGFP), and pNb_190_-mKO produces fusion proteins of nanobody with monomeric Kusabira Orange (mKO). Human epithelial HeLa cells and human embryonal 293T cells were maintained using standard procedures. For transfection of plasmid DNA, HeLa cells were plated onto glass bottom micro-well dishes (MatTek corporation) at 0.25×10^6^ cells/plate and cultured until 50% confluent. The cells were washed with PBS and transfected with plasmid DNA using SuperFect transfection reagent (Qiagen) according to the manufacturer’s manual and incubated overnight. 293T cells were cultured in micro well dishes until 50% confluence and transfected by the calcium phosphate co-precipitation technique. The NL4-3 molecular clone has been described previously [23]. Mutations for the alanine scan of Nb_190_ were obtained by mutating every residue (except for glycine residues) in the three hyper- variable nanobody domains to an alanine by the Gene Tailor Site-Directed Mutagenesis Kit (Invitrogen). For the virus expression experiments typically 0.5 µg of pNL4-3 and 1 µg of pcDNA3.1-Nb_190_ plasmids were used. Virus expression was analyzed by measuring the virus-associated core antigen (p24) in the supernatants of transfected cells by an enzyme-linked immunosorbent assay (GE Healtcare).

### Microscopy and Fluorescence Recovery after Photobleaching

Transfected HeLa cells were imaged with a laser-scanning SP5 confocal microscope (Leica Microsystems) equipped with an DMI 6000 microscope and an Acousto optical beam splitter, using an HCX plan apochromat x63 (numerical aperture 1.2) water immersion objective magnification. AcGFP was monitored with the argon laser using the 488-nm line for excitation, and emission was detected between 495 nm and 550 nm. mKO was imaged using the DPSS 561-nm laser for excitation, and emission was detected between 570 nm and 670 nm. Fluorescence recovery after photobleaching (FRAP) studies were performed by obtaining a series of 20 pre-bleach images of a nucleolus at 5% Acousto optical tunable filter (AOTF) laser setting, followed by a single 0.39 s bleach iteration at full laser power of a 1 µm wide circle in the nucleolus. Subsequent post-bleach images were acquired at 5% AOTF with the following time scheme: 20 images every 0.39 s, 20 images every 1 s, and 15–55 images every 10 s. Data were background-subtracted, bleach-corrected, and normalized according to the method described in [24]. Half-times of recovery were obtained by fitting the recovery curves employing GraphPad Prism (GraphPad Software, Inc.).

### Protein Expression and Purification

pET29b(+) and pET21b(+) constructs encoding respectively the Nb_190_ mutants and fusion proteins ECFP-Rev and EYFP-Rev were transformed in *E. coli* BL21(DE3), and expressed at 28°C (Nb_190_ mutants) or at 37°C (Rev fusion proteins) for 3.5 h in 1 mM isopropyl 1-thio-β-D-1-galactopyranoside (IPTG). Cells were lysed by sonication using the microson ultrasonic cell disruptor (Misonix). Proteins were purified *via* Ni^2+^-nitrilotriacetic acid affinity chromatography and stored at −20°C in 50 mM Tris-HCl (pH 7.8), 500 mM NaCl.

### Rev Multimerization Assay

The FRET-based Rev multimerization assay was performed as previously described [25]. Briefly 0.1 µM ECFP-Rev and 0.2 µM EYFP-Rev fusion proteins were mixed and serial 1/2 dilutions of Nb_190_ mutants were added starting from 1.5 µM. After a 30 min incubation FRET was determined using the Safire^2^ spectrofluorometer (Tecan). Emission was measured at 476±5 nm and 528±5 nm after excitation with 430±5 nm and at 528±5 nm after excitation with 490±5 nm. From these data the FRET efficiency was calculated according to the formulas described in [25].

### Mobility Shift Assays

High-affinity IIB hairpin RRE labelled with Alexa Fluor 633 was purchased from Sigma Aldrich. Proper secondary structure was obtained by diluting the RNA to 100 nM in buffer containing 10 mM Tris-HCl pH 7.8, 100 mM NaCl followed by heating to 95°C and stepwise cooling in a heat block. Binding reactions of Rev to RNA were performed in buffer containing 20 mM Tris-HCl (pH 7.8), 100 mM NaCl, 10 mM ditriothreitol with 0.1 mg/ml bakers’ yeast tRNA and 0.1 mg/ml bovine serum albumin. Typically, 5 nM of RRE, 60 nM of Rev protein and 300 nM of Nb_190_ mutants were used. The samples were incubated for 20 min at room temperature and run on a 6% polyacrylamide gel for 1 h. The bands were visualized and quantified using an Ettan™ DIGE Imager (GE Healthare).

### Amino Acid Conservation Model of Nb_190_

Amino acid conservation of Nb_190_ was calculated using the Consurf server [26]. This server compares the sequence of Nb_190_ with homologous crystallized proteins to detect conserved residues.

### Nb_190_ Homology Model

A homology model of Nb_190_ was constructed using the antibody modeler tool in MOE (Chemical Computing Group, Montreal, Canada). CDR loop grafting was performed to enhance the reliability of the template in the loop regions [27]. In addition to sequence identity, the selection of templates in MOE is based on a structure score, which is calculated by assessing the backbone integrity of the F_V_ dimer with global structure factors (such as resolution, R-value and R_free_ value) and local backbone investigation (topology, geometry, B-factors and occupancy of the residues). The structure score of a template can vary from 0 (not appropriate for homology modeling) to 100 (perfect template). The PDB structure 3EZJ was used as the main template, which has very good overall sequence identity (82.1%). In addition, the PDB structure 1OAR was used as template for the CDR3 loop, since it has the highest MOE structure factor in this specific loop fragment (94.4). Twenty-five models based on the combination of five main chain models with five side chain models for each main chain were constructed. The best model according to the GB/VI scoring function [28], [29] was further refined using energy minimization with the Amber99 force field [30].

### Docking of Nb_190_ to Rev

To improve the docking procedure, an ensemble of starting structures was generated using Molecular Dynamics (MD) on both the Rev protein and the nanobody. For the Rev protein, the 3LPH tetrameric crystal structure was converted to the wild-type sequence (mutations S12L and R60L) and prepared as described previously [31]. Chains C and D of the tetramer were omitted to obtain only one dimer (chains A and B). This dimer, containing two multimerization sites, was simulated with the Gromacs package (version 4.5.3) for 10 ns [32]. Next the trajectory was fitted on the multimerization binding site of chain B (comprising D9 to Y23 and I52 to Y63). The structures of monomer B in this trajectory were subsequently clustered using the Jarvis-Patrick algorithm [33]. Twelve clusters were found and one central structure in each cluster was written out as a PDB-file. For the nanobody the homology model was used as input structure and simulated for 10 ns as well. However, the fitting of the trajectory was performed on the backbone of the secondary structure elements, while clustering was conducted on differences in the CDR-loops to take into account the flexibility of these loop conformations. In total thirteen clusters were obtained.

Molecular docking of Nb_190_ onto Rev was simulated using HADDOCK (version 2.0) [34]. As explained above, an equilibrated ensemble of clustered structures extracted from MD-simulations were used as starting structure for the docking protocol. The following active residues were defined based on the experimental alanine scanning information: K20 and Y23 in the Rev protein and T33, F100, and Y105 in Nb_190_. Amino acids of secondary importance were defined as passive residues: V16, H53 and L60 in the Rev protein and D107 in Nb_190_. Three nanobody residues F50, N96 and D98 were neglected because in preliminary docking experiments they turned out to be important for Nb_190_ structure, rather than direct Nb_190_-Rev contacts. During the docking phase, residues 98 to 107 of the nanobody were set as semi-flexible to allow relaxation of the CDR3 loop. The final docked conformations were clustered based on the interactions of K20 and Y23 in the Rev protein with the active residues of Nb_190_ and inspected visually. Since both K20 and Y23 are crucial for the interaction of Rev with Nb_190_, models where only one of these residues made contacts with Nb_190_ were not taken into account.

## Results

### Evaluation of Nb_190_ Mutants in a Nb_190_-Rev Co-localization Assay

**Figure 2:**
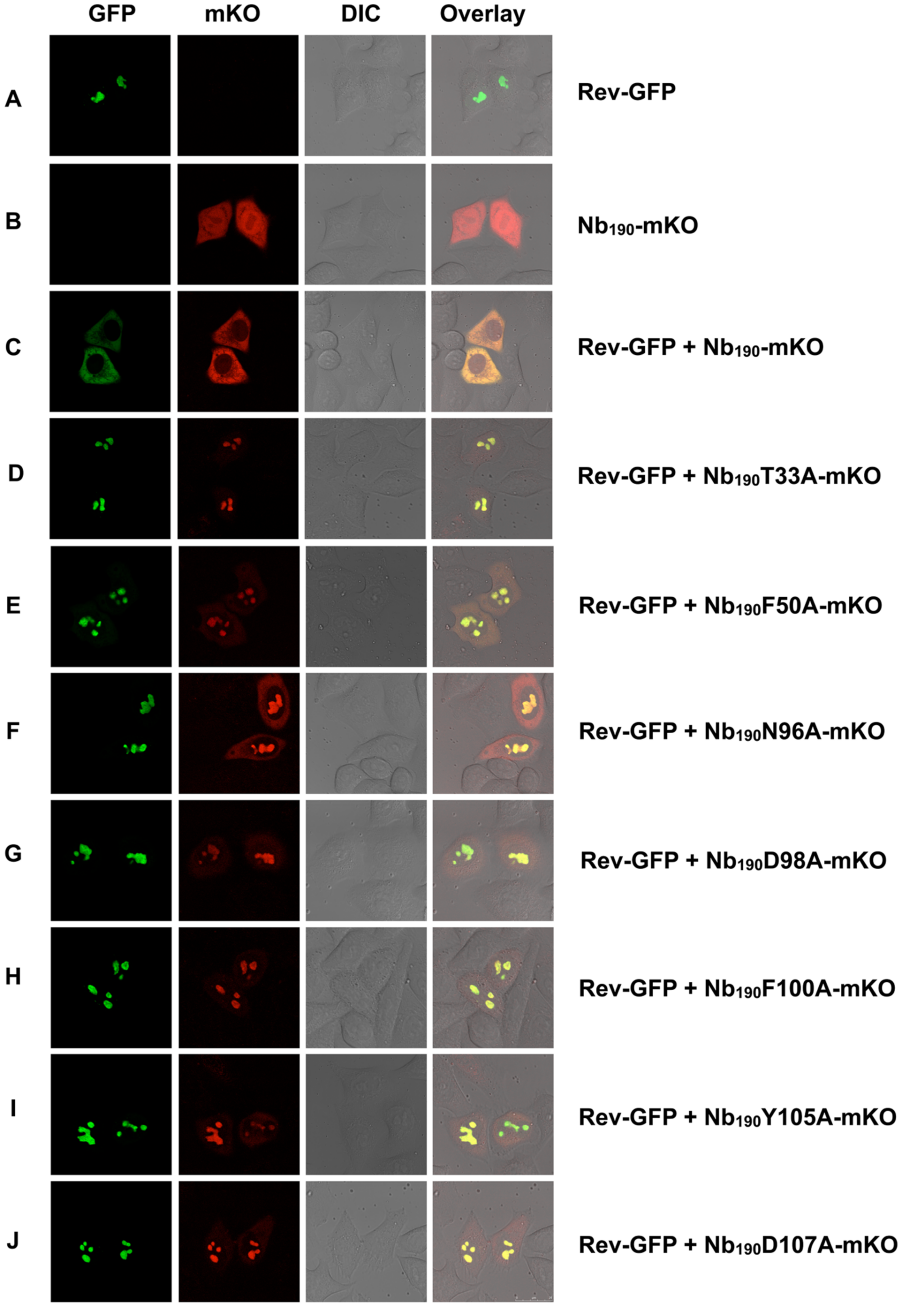
Nb_190_-Rev co-localization assay. HeLa cells were co-transfected with Rev-GFP and Nb_190_-mKO mutants expressing plasmids as indicated. Sub-cellular localization of the protein was visualized by confocal fluorescence microscopy in both GFP and mKO channels. The right column shows overlay images. DIC, differential interference contrast. (**A**) Rev-GFP localizes in the nucleoli. (**B**) Wild-type Nb_190_-mKO localizes throughout the cell, but not in the nucleoli. (**C**) Upon co-transfection, Rev-GFP and wild-type Nb_190_-mKO co-localize in the cytoplasm. (**D–J**) Seven out of the thirty-one Nb_190_-mKO alanine mutants co-localize with Rev-GFP in the nucleoli.

To obtain a detailed view on the Nb_190_ paratope, we set up an alanine scan on the three hyper-variable domains of Nb_190_. All residues in these CDR domains (except for glycine) were mutated one by one to alanine (Fig. 1*B*). In a first approach these thirty-one alanine mutants of Nb_190_ were evaluated in a Nb_190_-Rev co-localization assay as described previously in [20]. The Rev-GFP fusion protein expressed from a transfected plasmid localizes mainly to the nucleoli (Fig. 2A), while wild-type Nb_190_ fused to mKO is found both in the cytoplasm and the nucleus, but is excluded from the nucleoli (Fig. 2*B*). When these two proteins are co-expressed in the same cell, Rev-GFP and Nb_190_-mKO co-localize in the cytoplasm (Fig. 2*C*), implying interaction between these two proteins. Twenty-four of the thirty-one alanine mutants showed a localization similar to wild-type Nb_190_ (data not shown). Seven mutants (T33A, F50A, N96A, D98A, F100A, Y105A and D107A) were hardly found in the cytoplasm, but co-localized with Rev-GFP in the nucleoli (Fig. 2*D*-2*J*). This result illustrates that these mutants still interact with Rev but do not cause Rev to localize in the cytoplasm as wild-type nanobody does.

### Affinity Measurement of the Selected Nb_190_ Mutants by FRAP

**Figure 3:**
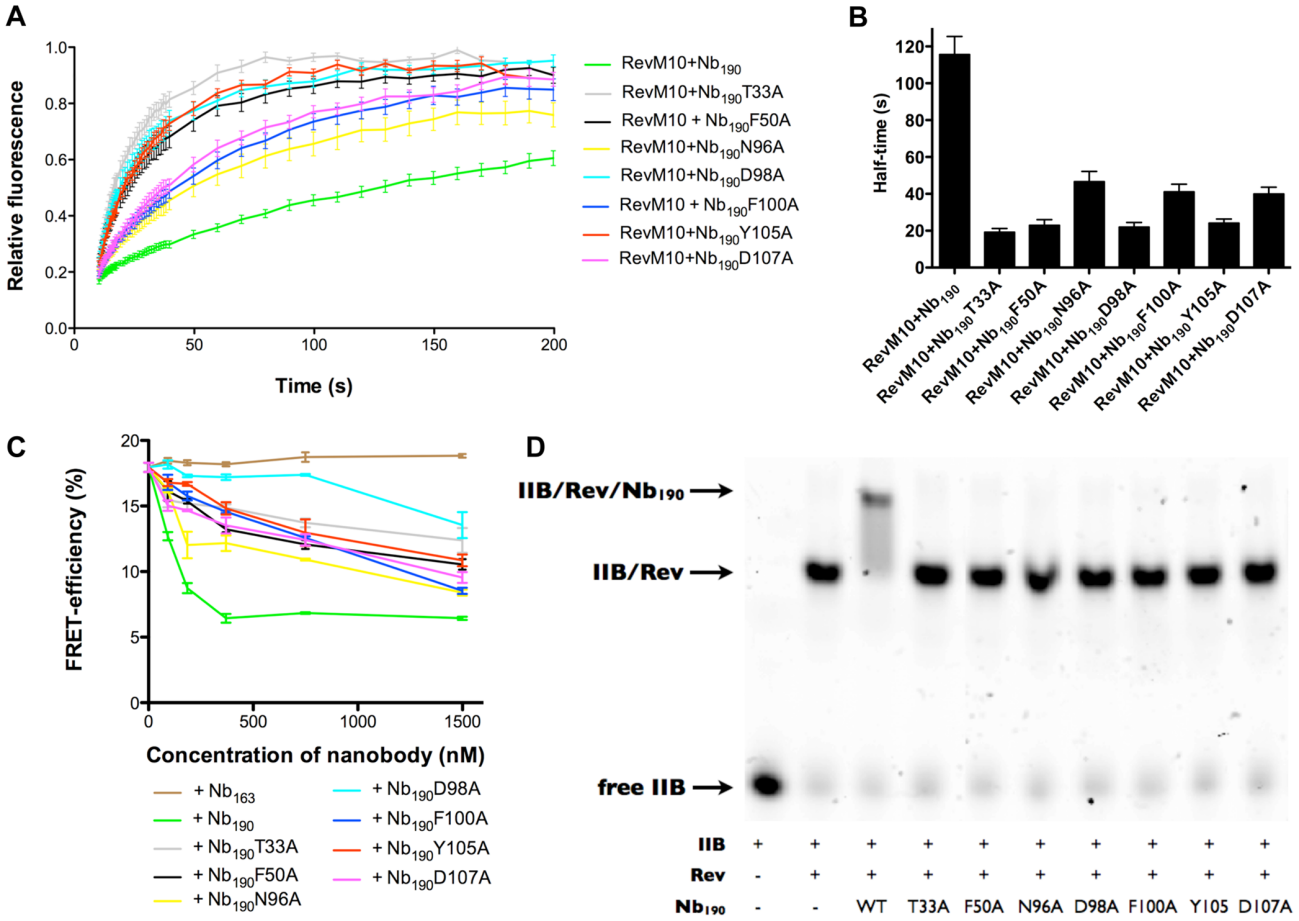
Relative affinity of the selected Nb_190_ mutants for Rev. (**A**) Fluorescence Recovery After Photobleaching (FRAP) of the Nb_190_-Rev interaction. Relative affinity of the selected Nb_190_ mutants was determined by FRAP of Nb_190_-mKO when bound to RevM10-GFP. Values are averages ± SEM (n ≥11). (**B**) Half-time values for the recovery times of Nb_190_ mutants after photobleaching. (**C**) Evaluation of the selected Nb_190_ mutants in the *in vitro* FRET multimerization assay. An ECFP-Rev/EYFP-Rev FRET sample is mixed with increasing amounts of Nb_190_ mutants. The Rev naive Nb_163_ is used as negative control. FRET efficiencies are presented as mean ± SEM (n = 3). (**D**) Evaluation of the selected Nb_190_ mutants by gel retardation. Gel mobility shifts of labeled high affinity stem IIB RRE RNA in complex with Rev were performed in the presence of Nb_190_ mutants. Bands corresponding to free high affinity binding stem IIB RRE (*lane 1*), IIB-Rev (*lane 2*), and IIB-Nb_190_-Rev complexes (*lanes 3–10*) are indicated.

To further address this observation, we tested whether the seven selected nanobody mutants have a reduced affinity for Rev in cells. Nb_190_-Rev interaction in living cells was therefore determined by fluorescence recovery after photobleaching (FRAP). As demonstrated above wild-type Nb_190_ causes Rev to localize in the cytoplasm, which makes affinity measurements using FRAP impossible. Therefore we employed a RevM10 mutant that is not exported to the cytoplasm, but remains in the nucleoli even in the presence of Nb_190_-mKO. The RevM10 mutation renders Rev nuclear export-deficient [35] and relatively immobile, without interfering with Nb_190_-Rev interaction. In this way, wild-type Nb_190_ co-localizes with RevM10 in the nucleoli similarly to the selected mutants and affinities of these mutants can be compared relative to the wild-type nanobody. For the seven selected Nb_190_-mKO mutants, recovery after photobleaching was much faster than for wild-type Nb_190_-mKO, which is indicative of a weaker interaction with Rev (Fig. 3*A and B*). Together, these FRAP data imply that the altered co-localization from cytoplasmic to nucleolar of the Nb_190_ mutants with Rev as compared to wild-type Nb_190_ correlates with a reduced affinity for Rev.

### Affinity of the Selected Nb_190_ Mutants for Rev *in Vitro*

Next we aimed at confirming the obtained results in two *in vitro* Nb_190_-Rev affinity assays. The seven selected nanobody mutants and wild-type Nb_190_ were therefore expressed and purified from *E. coli*. First we assessed their ability to inhibit *in vitro* Rev-Rev interactions in a fluorescence resonance energy transfer (FRET)-based Rev multimerization assay [25]. In this experiment interaction between ECFP-Rev and EYFP-Rev fusion proteins leads to an energy transfer from ECFP to EYFP. In the presence of wild-type Nb_190_ this FRET signal is dose-dependently inhibited, indicative of reduced Rev-Rev interactions. The Nb_190_ mutants were compared to wild-type (Fig. 3*C*). As expected, they all have a reduced ability to interfere with Rev multimerization. Although we could not observe a strict correlation between the FRAP and the FRET assay, the four mutants with the least affinity for Rev as measured by FRAP (Fig. 3*A*) were also the least active for inhibition of Rev multimerization (Fig. 3*C*).

To test whether the Nb_190_ mutants were still able to interact with Rev that is bound to Rev-specific RNA, a gel-mobility shift assay was performed. We incubated Rev with the labeled IIB RRE stem-loop and wild-type Nb_190_ or the mutant nanobodies. Wild-type Nb_190_ binds Rev when the latter is associated with the IIB high affinity loop of RRE as demonstrated by the super shift in *lane 3* of Fig. 3*D*. None of the seven mutants caused such a shift. Overall, we conclude that the selected mutants have a reduced affinity for Rev, a reduced capacity of blocking Rev-Rev interactions *in vitro* and that they cannot bind to IIB associated Rev anymore, all in agreement with a reduced affinity of these mutants for Rev in living cells.

### Antiviral Effect of the Nb_190_ Mutants on HIV-1 Production

**Figure 4:**
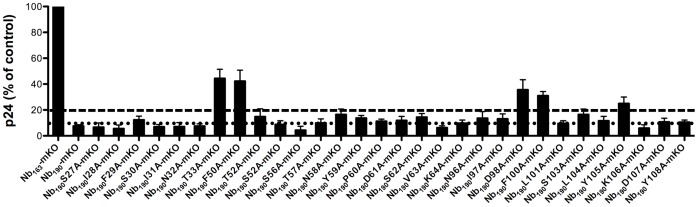
Inhibition of the Nb_190_ mutants in an antiviral assay. 293T cells were co-transfected with the pNL4-3 molecular clone (0.5 µg) and plasmids expressing Nb_190_ mutants (1 µg). Production of virus was analyzed by quantifying the virus-associated core antigen (Gag p24) in the supernatants of the transfected cells. Values are expressed as percentages relative to the p24 expression levels of cells transfected with pNL4-3 and a plasmid expressing the Rev naive Nb_163_. 80% and 90% inhibition of HIV-1 production is indicated respectively by lines –– and …… Results are mean ± SEM (n = 3).

Thus far, we have evaluated the Nb_190_ alanine mutants in several Nb_190_-Rev interaction assays. In a second approach, we assessed the ability of these mutants to inhibit HIV-1 replication. Plasmids expressing the Nb_190_ alanine mutants were co-transfected with an NL4-3 viral plasmid and after 24 hours HIV-1 virus production was measured by quantifying the virus associated p24 core antigen in the supernatant by ELISA (Fig. 4). Wild-type Nb_190_ reduced the HIV-1 production more than 90% compared to the Rev naive nanobody Nb_163_, in agreement with earlier results [20]. Mutants T33A, F50A, D98A, F100A and Y105A, but not N96A and D107A reduced this p24 level less than 80%, suggesting that these residues play an important role in the Nb_190_ mediated inhibition of HIV-1 production. These results also largely confirm the observed lower affinity of the nanobody mutants for Rev.

### Mapping of the Rev epitope

**Figure 5:**
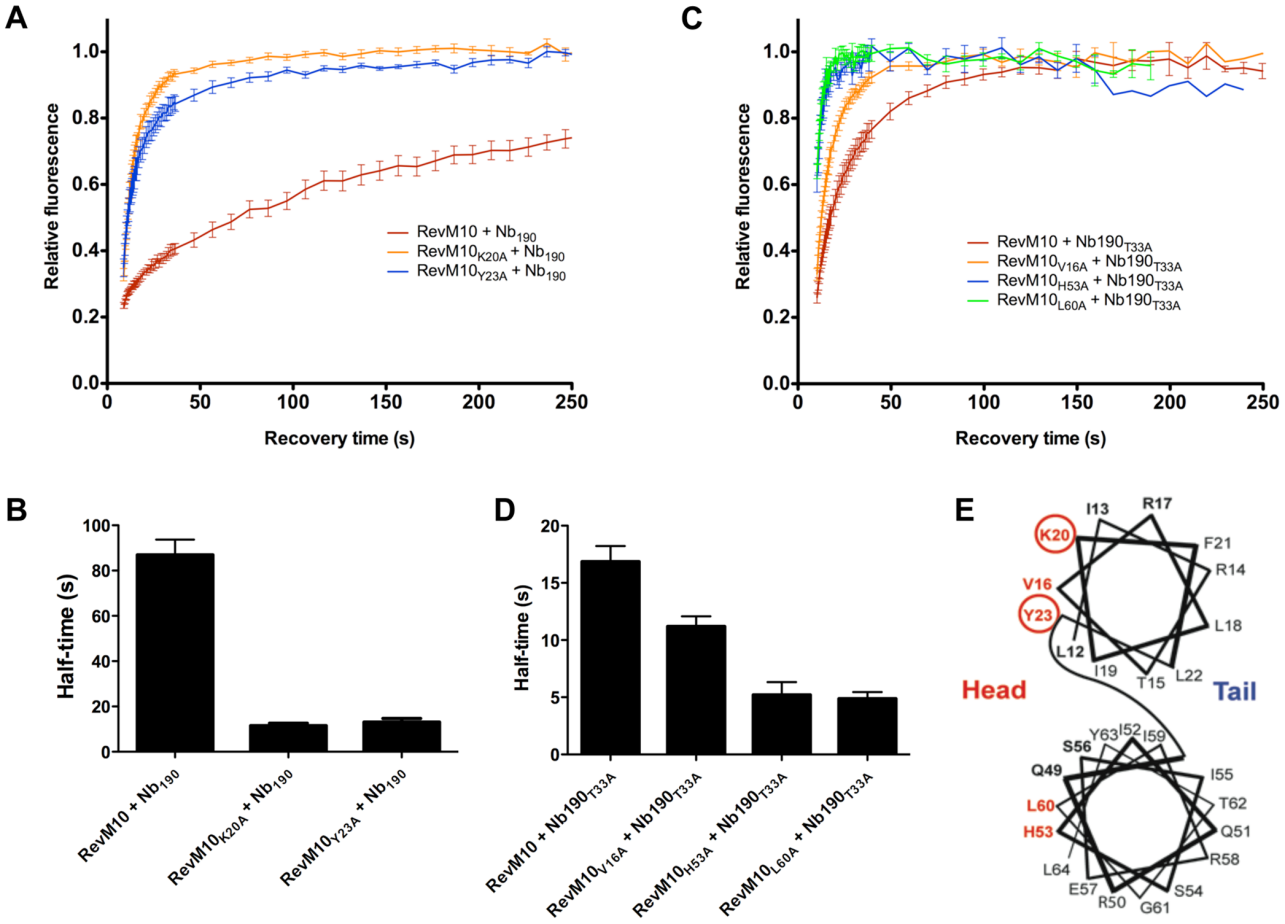
Mapping of the Rev epitope. (**A**) Relative affinity of wild-type Nb_190_ for the K20A and Y23A Rev mutants by FRAP of Nb_190_-mKO when bound to RevM10-GFP. Values are averages ± SEM (n ≥8). (**B**) Half-time values for the recovery times after photobleaching in panel A. (**C**) Relative affinity of Nb_190_T33A for the V16A, H53A and L60A Rev mutants by FRAP of Nb_190_-mKO when bound to RevM10-GFP. Values are averages ± SEM (n ≥6). (**D**) Half-time values for the recovery times after photobleaching in panel C. (**E**) Schematic overview of the alanine scan performed on the head multimerization surface of Rev. Residues that were mutated to alanine are shown in bold. Mutated positions that resulted in a decreased affinity for the Nb_190_T33A mutant are shown in bold red. Mutated positions that resulted in a decreased affinity for the wild-type nanobody have a red circle.

Previously, we have shown that Nb_190_ binds the N-terminal head multimerization surface of Rev [20]. To map the Rev epitope in more detail, we therefore also performed an alanine scan on the residues in this domain. Two of the eleven residues (K20A and Y23A) gave a significant different affinity of wild-type Nb_190_ for the Rev mutants in Nb_190_-Rev FRAP experiments, demonstrating their importance for epitope recognition (Fig. 5*A and B*). However, a protein-protein interaction surface usually contains more than two residues. Due to the high affinity of wild-type Nb_190_ for Rev, it might be possible that subtle changes in Rev affinity were not detected. Therefore we used an attenuated nanobody carrying the T33A mutation that was identified above. When the eleven Rev mutants were combined with the T33A mutation in Nb_190_, three extra Rev residues were found to affect the affinity of the nanobody for Rev, resulting in a lower half-time for recovery after photobleaching than wild-type Rev: V16, H53 and L60 (Fig. 5*C and D*). In conclusion, from the eleven residues in the N-terminal head multimerization surface of Rev we selected two residues (K20 en Y23) that are crucial for the Nb_190_-Rev interaction and three residues (V16, H53 and L60) that contribute to this interaction to lesser extent (Fig. 5*E*).

### Molecular Docking of Nb_190_ to Rev

**Figure 6:**
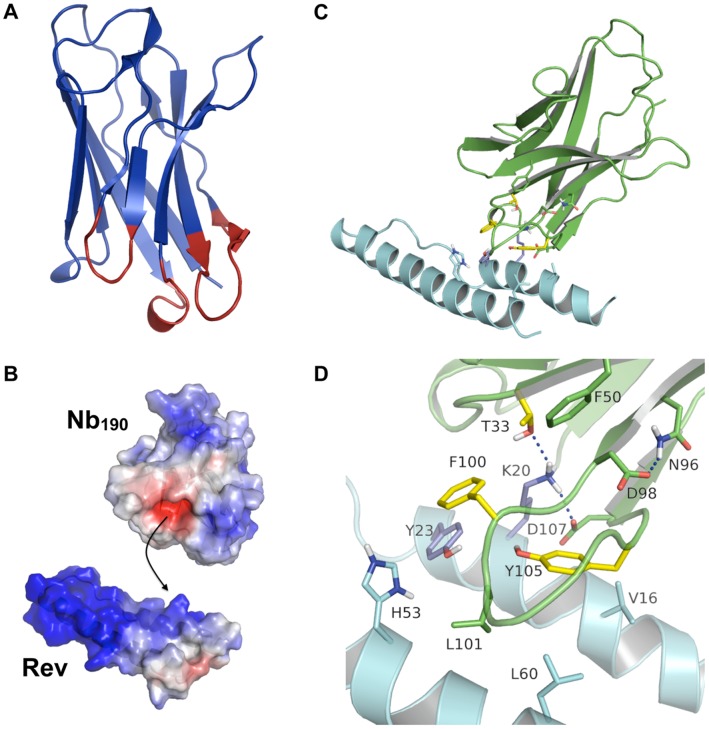
Nb_190_-Rev interaction model. (**A**) Cartoon representation of the Nb_190_ homology model. The coloring corresponds to the conservation of the amino acids calculated with the Consurf server, ranging from highly variable residues (red), over intermediate conservation (green), to highly conserved residues (blue). (**B**) Electrostatic surface of Nb_190_ and helix-tun-helix motif of Rev: blue (negative), red (positive) and white (neutral). The interaction between Nb_190_ and Rev is a combination of electrostatic interactions between RevK20 and the negative pocket of Nb_190_ and further hydrophobic stabilization by the surrounding residues. (**C**) Cartoon representation of Nb_190_ (green) docked onto Rev (N-terminal helix-turn-helix motive) (light blue). Important residues for binding interaction according to the alanine scanning results are colored dark blue for Rev and yellow for Nb_190_.). Aliphatic hydrogens and backbone atoms have been hidden for clarity. (**D**) Close-up of the Nb_190_-Rev interaction pattern, with residue RevK20 forming an extensive hydrogen bonding network (depicted by blue lines) with neighboring residues T33 and D107 in Nb_190_. In addition, RevY23 makes a π-π interaction with Nb_190_F100. This latter residue is also further stabilized by hydrophobic contacts with RevK20 and RevH53. RevH53 and RevL60 interact with Nb_190_L101, while RevV16 makes contact with Nb_190_Y105. Nb_190_D98 stabilizes the CDR3 loop by hydrogen bonds with Nb_190_N96.

The obtained mutational and functional data enabled us to draw a detailed Nb_190_-Rev interaction model. Because no structure for Nb_190_ is available yet, a homology model was first constructed using the MOE software package. Fig. 6*A* displays the secondary structure, which is very similar to resolved crystal structures of other nanobodies such as 3EZJ and 1SJX. Using the Consurf server [26], we compared the Nb_190_ sequence to 150 other nanobody sequences and determined which amino acids are the least conserved. In Figure 6*A* the homology model residues of Nb_190_ are colored according to the conservation grade with CDR3 displaying the highest variability, followed by CDR1 and CDR2. If we compare this model to our mutational data, we find that five of the seven selected amino acid positions (T33, F50, D98, F100 and Y105) are part of highly variable regions (red), while two positions (N96A, D107A) are only moderately variable (green).

Based on our detailed epitope and paratope mapping, the homology model of Nb_190_ was docked onto a Rev monomer. The following positions were considered hereby as crucial: K20 and Y23 in Rev and T33, F100 and Y105 in Nb_190_. Residues that are involved in Nb_190_-Rev interaction, but seem to be of minor importance are D107 in Nb_190_ and V16, H53 and L60 in Rev. Three selected residues from Nb_190_ were neglected based on preliminary scans in which we found them to be important rather for structural reasons and internal nanobody stabilization than for direct Nb_190_-Rev contacts: F50, N96 and D98. As shown in Fig. 6*B*, close contacts between Nb_190_ and Rev are mainly exerted by CDR3. The Nb_190_-Rev interaction is based on a central polar interaction, that surrounded by hydrophobic stabilizing contacts (Fig. 6*C*).

Fig. 6*D* shows a closer look of the Nb_190_-Rev interface. RevK20 makes strong polar interactions with Nb_190_T33, Nb_190_Y105 and Nb_190_D107. Interestingly, experimental data demonstrated that RevK20 is the most important Rev residue for interaction with Nb_190_ [20], while the T33 mutation of Nb_190_ generally shows the strongest reduction in Rev affinity and antiviral activity. Thus, this model supports the experimental data as highlighted by the importance of both residues. Furthermore, RevY23 makes a π-π interaction with Nb_190_F100. The polar centre of the interaction surface is surrounded by hydrophobic residues in both Rev and Nb_190_. The F100 residue of the nanobody is further stabilized by hydrophobic contacts with RevK20 and RevH53. RevH53 and RevL60 mainly interact with Nb_190_L101, while RevV16 makes contacts with Nb_190_Y105. Residues N96 and D98 within Nb_190_ make hydrogen bonds with each other in order to stabilize the CDR3 loop. Based on the biological data Nb_190_F50 is also important for epitope recognition. This seems however not to be further supported by the Nb_190_-Rev interaction model, since the F50 residue makes no direct contacts with Rev. However, this residue possibly disrupts the nanobody β-sheet backbone structure or weakens the stability of the neighboring CDR3 loop. Overall this model elucidates the results from our biological assays and gives us new insights about the Nb_190_-Rev interface.

## Discussion

Recently we have identified a llama single-domain antibody (nanobody) against Rev. This intrabody (Nb_190_) binds the head multimerization surface of Rev and prevents Rev-Rev interactions. A similar approach using a monoclonal Fab fragment that binds the tail multimerization surface of Rev has been used to perform an antibody-aided crystallization of Rev [19]. Interestingly, Nb_190_ can easily be expressed inside mammalian cells and efficiently inhibits HIV-1 viral production in cell culture, representing the first Rev multimerization inhibitor with antiviral activity [20]. To gain further insight into the binding interface between Rev and Nb_190_, we mapped the nanobody paratope and the Rev epitope by performing mutational analysis on respectively the three hyper-variable regions of Nb_190_ and the N-terminal helix-turn-helix domain of Rev.

Seven out of the thirty-one Nb_190_ alanine mutants (T33A, F50A, N96A, D98A, F100A, Y105A and D107A) displayed a Nb_190_-Rev co-localization pattern different from wild-type. In contrast to cytoplasmic co-localization, these mutants co-localized with Rev in the nucleoli, which correlated with a lower affinity for Rev as measured in three other Nb_190_-Rev interaction assays. Five out of the seven identified mutants also had a significant lower ability to inhibit HIV-1 production.

Wild-type Nb_190_ causes Rev to accumulate in the cytoplasm, while it is still able to shuttle to the nucleus [20]. This observation raises the question whether instead of blocking of Rev-Rev interactions, the inhibitory effect of Nb_190_ could be due to sequestration of Rev to a wrong sub-cellular localization. Our results demonstrate this is, at least in part, not the case as the Nb_190_ mutants do not sequester Rev to the cytoplasm and co-localize with Rev in the nucleoli (Fig. 2), while retaining considerable antiviral activity (Fig. 4). Moreover, the cytoplasmic distribution of Rev caused by the presence of wild-type Nb_190_ is similar to the distribution caused by mutations that impair the multimerization of Rev [20]. Nb_190_ mutants with lower affinity for Rev also display a decreased inhibition of Rev multimerization (Fig. 3) and therefore most likely do not provoke the cytoplasmic distribution which is observed upon inhibition of Rev multimerization. In our previous publications, we showed that Nb_190_ binds to the head multimerization surface and inhibits Rev multimerization *in vitro* both with and without Rev specific RNA [20], [25]. Although we also demonstrated that Nb_190_ inhibits Rev-Rev interactions in cell culture, we cannot rule out the possibility that in a viral context additional crucial Rev functions are impaired by the nanobody. The observation that N96A and D107A mutants show reduced inhibition in the *in vitro* Rev multimerization assay, while retaining considerable antiviral activity, could indeed imply that additional Rev functions are impaired and is the subject of further ongoing research.

Several DEAD box helicases have been identified as cellular cofactors for Rev [36], [37]. DDX1 has been identified by yeast two-hybrid screening with the first Rev multimerization domain as bait and acts as a co-factor of Rev [38]. Although the precise interactions of DDX1 with Rev are unknown, the Nb_190_-Rev binding site in our model clearly overlaps with the Rev-DDX1 contact region, suggesting that Nb_190_ might disrupt this interaction. The Nb_190_-Rev cytoplasmic co-localization further supports this idea because impaired HIV-1 Rev function in astrocytes is also associated with a cytoplasmic accumulation of Rev [39], [40], which could be linked to downregulation of DDX1 [36]. Moreover, DDX1 has recently been shown to stimulate Rev multimerization *in vitro* [41]. Therefore, inhibition of HIV-1 by Nb_190_ could be a direct effect on Rev-Rev interactions, combined with an indirect effect *via* DDX1.

Since the discovery of HIV-1 Rev protein as being crucial for viral RNA export and replication, many efforts have been made to propose Rev function as a new target for antiviral therapy. None of the identified Rev inhibitors made it into clinical trials yet. The small protein Nb_190_ is the first Rev inhibitor interacting with the N-terminal multimerization domains, hereby efficiently inhibiting HIV-1 replication. One of the possible applications for Nb_190_ could be its direct use in gene therapy, although this field is still in full development. Today, most of the existing HIV-1 therapies target the viral enzymes reverse-transcriptase, integrase or protease. Active sites of enzymes form relatively small cavities/clefts shielded from the solvent *via* hydrophobic contacts. In contrast to protein-protein interactions, enzyme substrates can often serve as template for the design of enzyme inhibitors. However, many of the essential steps of the HIV-1 replication cycle require protein-protein interactions like Rev multimerization or these of viral proteins with cellular co-factors. Compared to the 300–1000 Å interaction interface between small molecules and proteins, protein-protein interactions typically have a 1500–3000 Å buried interface, which is often non-contiguous and relatively featureless [42]. Although it has been thought for years that protein-protein interactions are not a valuable target for drug development, several classes of HIV-1 inhibitors of these interactions have been discovered [43]–[47]. The structural Nb_190_-Rev interaction model proposed here could serve the development of a pharmacophore model used for the rational design of small-molecule inhibitors of the Rev N-terminal domain protein-protein interactions. A similar strategy has been used to target several other protein-protein interactions, including MDM2:p53 [48], Bak BH3:Bcl2/Bcl-XL [49] and the HIV-1 integrase LEDGF/p75 complex [44]. In addition, this nanobody proves to be a great molecular tool to investigate the Rev multimerization mechanism and other functions of the Rev-dependent nucleocytoplasmic transport.
